# First clinical applications for the NIR-II imaging with ICG in microsurgery

**DOI:** 10.3389/fbioe.2022.1042546

**Published:** 2022-10-18

**Authors:** Yifan Wu, Yongkuan Suo, Zheng Wang, Yifeng Yu, Shuang Duan, Hongguang Liu, Baiwen Qi, Chao Jian, Xiang Hu, Dong Zhang, Aixi Yu, Zhen Cheng

**Affiliations:** ^1^ Department of Orthopedics Trauma and Microsurgery, Zhongnan Hospital of Wuhan University, Wuhan, China; ^2^ Joint Laboratory for Molecular Medicine, Institute of Molecular Medicine, Northeastern University, Shenyang, Liaoning, China; ^3^ State Key Laboratory of Drug Research, Molecular Imaging Center, Shanghai Institute of Materia Medica, Chinese Academy of Sciences, Shanghai, China; ^4^ Shandong Laboratory of Yantai Drug Discovery, Bohai Rim Advanced Research Institute for Drug Discovery, Yantai, Shandong, China

**Keywords:** indocyanine green (ICG), near-infrared II (NIR-II) imaging, fluorescenceguided surgery, vascular anastomosis, flap reconstruction

## Abstract

In microsurgery, it is always difficult to accurately identify the blood supply with ease, such as vascular anastomosis, digit replantation, skin avulsion reconstruction and flap transplantation. Near-infrared window I (NIR-I, 700—900 nm) imaging has many clinical applications, whereas near-infrared window II (NIR-II, 1,000–1700 nm) imaging has emerged as a highly promising novel optical imaging modality and used in a few clinical fields recently, especially its penetration distance and noninvasive characteristics coincide with the needs of microsurgery. Therefore, a portable NIR-II imaging instrument and the Food and Drug Administration (FDA) approved indocyanine green (ICG) were used to improve the operation efficiency in microsurgery of 39 patients in this study. The anastomotic vessels and the salvaged distal limbs were clearly visualized after intravenous injection of ICG. The technique enabled identification of perforator vessels and estimation of perforator areas prior to the flap obtention and made it easier to monitor the prognosis. Overall, this study highlights the use of the portable NIR- II imaging with ICG as an operative evaluation tool can enhance the safety and accuracy of microsurgery.

## Introduction

Vascular anastomosis is a common procedure used in various surgical subspecialties (Lee et al., 1980). In particular, it is a critical constituent of reconstructive microsurgery, vascular surgery, and transplant surgery. The conventional suture-based anastomosis technique has seen many technological advances from the improvement on microscopes, to improved suture material and non-suture cuff techniques (Lee et al., 1980; Zeebregts et al., 2003; Ma and Fei, 2021). However, quality assessment of the patency of anastomotic vessels and surgical outcomes depend merely on the experience of the surgeon, and an available standardized imaging technique is still absent.

Digit replantation is developed based on vascular anastomosis. Anastomosis failure and vascular congestion are the leading causes of replantation failure (Agarwal et al., 2018). The surgical techniques used, especially vascular anastomotic techniques, are the most important factors that determine the success of a replantation attempt. Anastomotic patency is usually assessed with “empty-and-refill” test. This involves first emptying a vascular segment with the use of 2 micro-forceps, removing the occluding forceps, and then observing the refilling velocity. High-quality vascular anastomosis is indicated by a “swift” refill (Ghanem et al., 2018). However, an explicit criterion of the valid refilling velocity is not available, because the evaluation was wildly based on the surgeon’s experience and personal judgement. On top of that, the complexity cause of trauma may lead to avulsion injury and vascular damage at the distal end of the digit. Even though the vascular anastomosis is successfully accomplished, there is still the hidden danger of insufficient distal blood supply for the replanted digit causing poor prognosis (Neuhaus et al., 2013; Diviti et al., 2017). In the field of imaging, an application providing immediate intraoperative microvascular anastomosis evaluation and far-end blood supply assessment is deemed to be the goal.

Skin avulsion injuries caused by high-energy traffic and machinery accidents are important topics in the field of repair and reconstruction. The injury generates a skin flap with uncertain vascular basis resulting in ischemic necrosis of the distal portion of the flap (Boettcher-Haberzeth and Schiestl, 2013). Currently, skin avulsion injury reconstruction is mainly based on subjective evaluation of traditional clinical signs. Yet there is lack of reliable approach for estimating the extent of blood supply in damaged tissue, which has frequently resulted in unnecessary tissue loss and incomplete debridement-related infection, limited the possibility of prompt surgical intervention.

Perforator flap reconstruction is also important in plastic and reconstructive surgery where vascular anastomosis is performed, prior to evaluate the patency of the vessels, the selection of a suitable perforator is critical for decreasing the rate of postoperative fat necrosis and minimizing perfusion-related flap complications (Clarke-Pearson and Kim, 2014; Kozusko et al., 2019). A thorough understanding of the vascular anatomy and flow characteristics of a selected perforator is essential to balance flap design and adequate flap perfusion. Technological advancements within imaging modalities have proved valuable in preoperative planning and intraoperative assessment.

Yet, an imaging modality that can accurately reflect the vessel circulation for microvasculature in a non-invasive, dynamic, real-time way remains urgently needed. Computed tomographic angiography (CTA) has become a commonly used technique for the preoperative locating and assessment of vessel circulation, however, the inconvenience that it cannot be used synchronously during the surgery still exists (Teunis et al., 2013; Fredrickson et al., 2017). Handheld Doppler devices are used both for preoperative and postoperative monitoring vessel circulation (Cheng et al., 2012), near-infrared window I (NIR-I, 700–900 nm) fluorescence imaging has also been introduced into the observation (Zhang et al., 2021b), but the resolution of these two methods is not always satisfactory.

Fluorescence imaging technique, characterized by high sensitivity, non-invasiveness and no radiation hazard, has been widely used in the biomedical field. To further improve the imaging quality of the NIR fluorescence imaging technique, near-infrared window II (NIR-II. 1,000–1700 nm) fluorescence imaging was developed recently, providing a highly versatile platform for non-invasive *in vivo* bioimaging to probe deeper into the biological tissues/organs with a greater degree of clarity (Hong et al., 2012; Chen et al., 2021; Li et al., 2021). Previously, our group have performed varies research with this technique along with a series of probes in animal models to study the physiological and pathological state of limb circulatory system.

In this study, we firstly used the portable NIR-II imaging instrument for imaging of 39 patients undergoing microvascular anastomosis, digit replantation, avulsion injury or perforator-based island flap surgery. The patients were injected with indocyanine green (ICG), which can be used for both NIR-I and NIR-II imaging and were approved by the Food and Drug Administration (FDA) for routine clinical use. Moreover, the imaging performance of the NIR-Ι window was compared with that of the NIR-ΙI window in each patient, to evaluate the potential of NIR-II image-guided microsurgery in clinic.

## Patient and methods

### Patient characteristics

This study was approved by the Medical Ethics Committee of Zhongnan Hospital of Wuhan University under protocol number 2021020. Patients were recruited from Zhongnan Hospital of Wuhan University in China between July 2021 and December 2021. Thirty-nine patients with an age of 18 years or older undergoing microvascular anastomosis, digit replantation, skin avulsion reconstruction and perforator-based island flap surgery were considered eligible for the study (detailed information is provided in the Supplementary Table S1). Informed consents of all patients were obtained. Exclusion criteria were patient experience of claustrophobia, pregnancy or lactation.

### NIR-I/II multispectral imaging instrument

**FIGURE 1:**
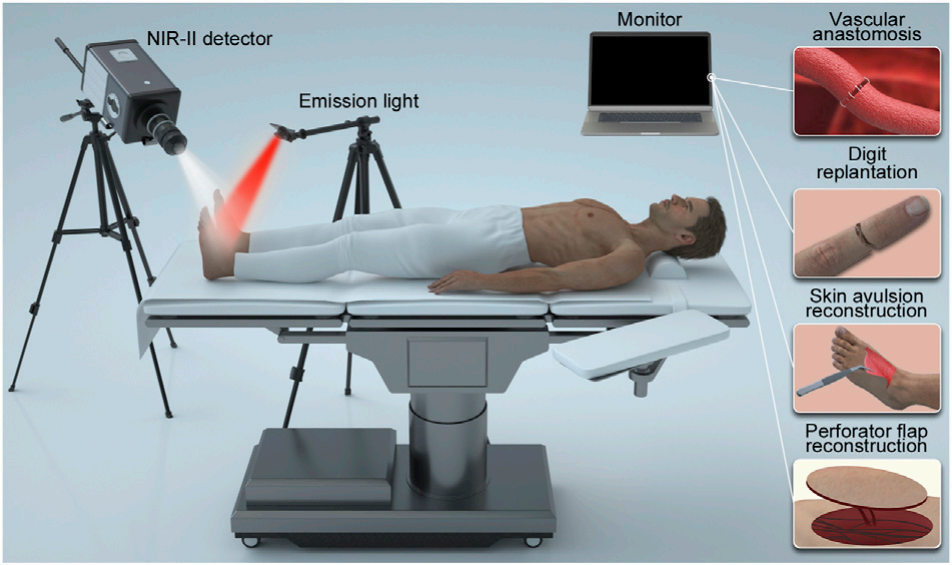
The illustration of the portable NIR-II imaging instrument in operation.

As shown in Figure 1, we constructed a NIR-II imaging system suitable for humans, covering the spectrum range of 900–1700 nm. The system is composed of an ultra-low temperature air cooling InGaAs camera (640 × 512 pixels, Hengxin, China) equipped with a prime lens (focal length: 50 mm, antireflection coating at 0.8–1.8 μm, Edmund Optics), cooled to −80°C.

Images in the NIR-I window were captured using a silicon camera (1920 × 1,080 pixels, ThorLabs, United States) equipped with a lens (focal length: 35 mm, ThorLabs, United States), which was fitted with a 800-nm long-pass filters and a 900-nm short pass filter to filter away 785 nm excitation and extract NIR-I fluorescence signal, respectively. A 785-nm laser device (Lasever, China) was used to provide uniform illumination on the field for NIR-I and NIR-II imaging synchronously. The facular power density was adjusted to 10–15 mW/cm^2^.

### Timing and ICG dosage

The fluorophore ICG was purchased from DanDong Pharmaceutical Factory (Liaoning, China). Details of the timing and ICG doses used, varying slightly according to each procedure, are described below.

### NIR imaging for assessing vascular patency during anastomosis

The study included 14 patients with open injury requiring microvascular anastomoses. The severed vessels included the superficial palmar arch, femoral, dorsalis pedis, posterior tibial, radial, ulnar, and popliteal arteries and posterior tibial vein. Two vascular clamps were placed in the distal and proximal parts of the broken artery. Anastomoses were performed in end-to-end fashion. After accomplishment of the anastomosis, a 2 ml of ICG was administered intravenously and NIR-I/II imaging were performed to assess the patency of arteries and veins. Direct examination using usual clinical patency tests (vessel filling and patency test) was also evaluated by the surgeon as objective controls of blood flow.

### NIR imaging for the evaluation of arterial supply and venous drainage during digital replantation

Eleven patients with amputated fingers requiring replantation were included. After initial debridement, anastomosis of artery and vein was performed. During operation, the number of artery and venous anastomoses determined by surgeons, using usual clinical tests (cutaneous bleeding, skin color and microcapillary refills). For severe damage, there was no suitable vein for direct anastomosis or bridging anastomosis. In this condition, only “bloodletting therapy,” a deliberate incision made on the belly of the distal digit, could be applied to alleviate the venous load, and venous drainage was re-established later during the wound healing. Once the anastomosis was completed, NIR-I/II imaging were performed with intravenous injection of 2 ml of ICG to evaluate the arterial blood supply and venous reflux. The velocity of fluorescence appearance and disappearance of the replanted digits was compared with those of the proximal normal fingers. Herein, the velocity of fluorescence appearance reflected the situation of arterial supply, while the velocity of fluorescence disappearance reflected the situation of venous drainage.

### NIR imaging for the evaluation of debridement (prediction of necrosis) during avulsion injury reconstruction

In this study, five patients with degloving injury were included. Before and after the first debridement (1, 3, 5, and 10 min), NIR-I/II imaging were performed with intravenous injection of 2 ml of ICG into the elbow vein of the patient to evaluate the arterial blood supply and venous reflux. To further verify the potential and accuracy of NIR-II fluorescence imaging in early prediction of skin necrosis in avulsion injuries, the status of avulsed skin was observed until post operation day 15. Second debridement was performed under the instruction by clinical observation and NIR imaging.

### NIR imaging for the detection of skin perforators and evaluation of flap perfusion

In this study, nine patients scheduled to undergo perforator-based island flap surgery for reconstruction of skin defects were included. Before performing NIR imaging, perforators were preliminarily located by Doppler sonography. Then, the flap was designed according to the location and size of the skin defect and marked on the skin with a felt-tipped pen. The patient was positioned with the affected site up, and the NIR-II imaging system was fixed at 20–40 cm above the skin. The NIR-I imaging system was placed in parallel with the NIR-II imaging system. When the marked possible perforator was in the territory of the target imaging region, 2 ml of ICG solution (2.5 mg/ml) was administered intravenously and fluorescent imaging was commenced synchronously. Based on the recorded images, the locations of possible perforators were marked on the skin with ink. During surgery, the perforators were detected, and it was confirmed whether the locations of the actual vessels corresponded with those predicted by NIR imaging. At the time of flap elevation, the presence of blood supply was assessed from the color of the flap skin and from bleeding at the margins. After the microvascular anastomoses were deemed open, the flap perfusion was assessed by clinical examination and with NIR imaging.

## Results

### NIR imaging for assessing vascular patency during anastomosis

**FIGURE 2:**
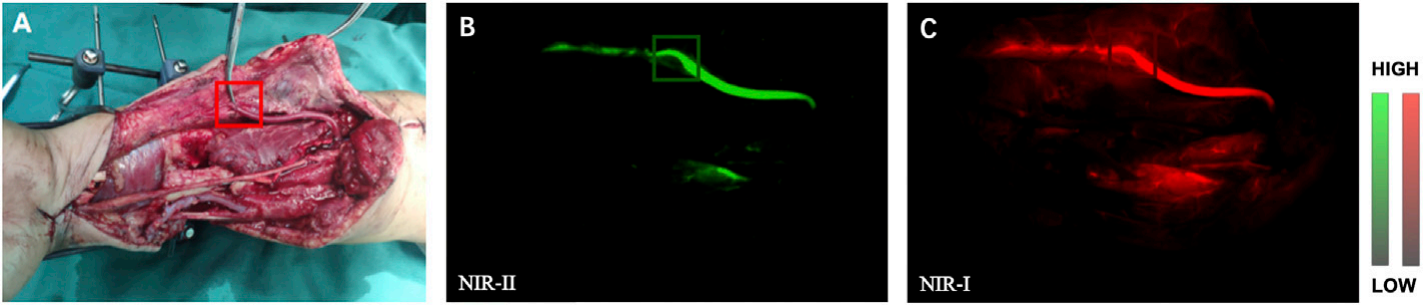
NIR Imaging for the assessment of vascular patency during anastomosis. **(A)** Intraoperative real-time views of vessel anastomosis. **(B)** Representative NIR-II window images for the assessment of artery patency. The blood quickly passed through the anastomosis. **(C)** Corresponding NIR-I window imaging.

The “patency test” confirmed successful anastomosis in all (14 patients) of the blood vessels. After the injection, blood flow within the proximal and distal areas of the vessel was rapidly visualized by NIR-I/II imaging (representative angiography shown in Figure 2), which was consistent with the results of “patency test.” However, NIR-I imaging showed obvious abundant circumjacent tissues and more background noise, while NIR-II imaging precisely showed the anastomotic vessels that needs to be tracked.

### NIR imaging for the evaluation of arterial blood supply and venous drainage during digital replantation

**FIGURE 3:**
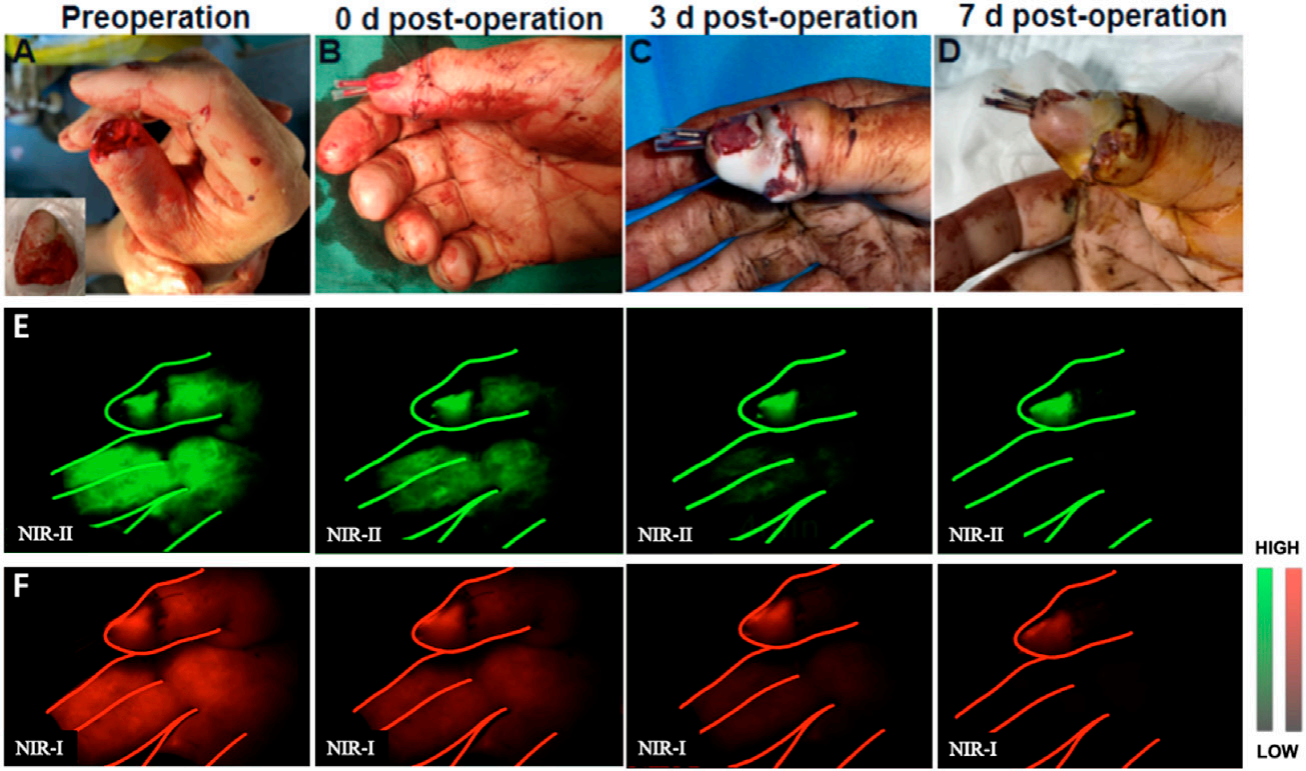
Example of a thumb replantation. **(A)** A 55-year-old man sustained complete amputation of the thumb. **(B)** The severed thumb digit was replanted. **(C,D)** View of replanted digit 3 and 7 days after initial injury. **(E,F)** Representative NIR-II and NIR-I window images for the assessment of arterial blood supply and venous reflux at different time points, respectively.

In our research, the replanted digits were completely fluoresced in all (11) of the patients without obviously delay after injection of 2 ml of ICG (representative angiography shown in Figure 3). And the fluorescence of normal digits completely disappeared within 5–8 min in both NIR-I and NIR-II imaging. No fluorescence disappearing velocity similar to that of normal finger was seen in all (10) of the patients. The fluorescence prolongation of replanted digits in four patients was 5–10 min longer than normal limbs. And the clinical tests including skin color, microcapillary refills of these replanted digits were normal. In addition, the fluorescence prolongation of replanted digits in four was 10–15 min longer than normal fingers. In these patients, the skin of the replanted digits appeared congestion and the velocity of microcapillary refills was considerably slower. Based on clinical tests, the surgeon did not increase the number of venous anastomoses, but chose to suture the wound sparsely, allowing for the stagnant venous blood seep out of the wound to relieve the venous load. Finally, the fluorescence prolongation of replanted digits in three patients was more than 15 min longer than normal fingers. Among them, two patients suffered from amputation of the end of thumbs. Although the veins were anastomosed, they might not be able to serve as a reliable backflow because the vessels were too thin. Thus, clinical examinations in operation also showed poor results, including swelling of the replantation digits and excessively bluish skin. For these patients, surgeons eventually decide to relieve the load of veins by bloodletting therapy. For another patient, surgeons chose to re-anastomose other veins. After 2 weeks of observation, all replanted digits eventually survived.

### NIR imaging for the evaluation of debridement (prediction of necrosis) during avulsion injury reconstruction

**FIGURE 4:**
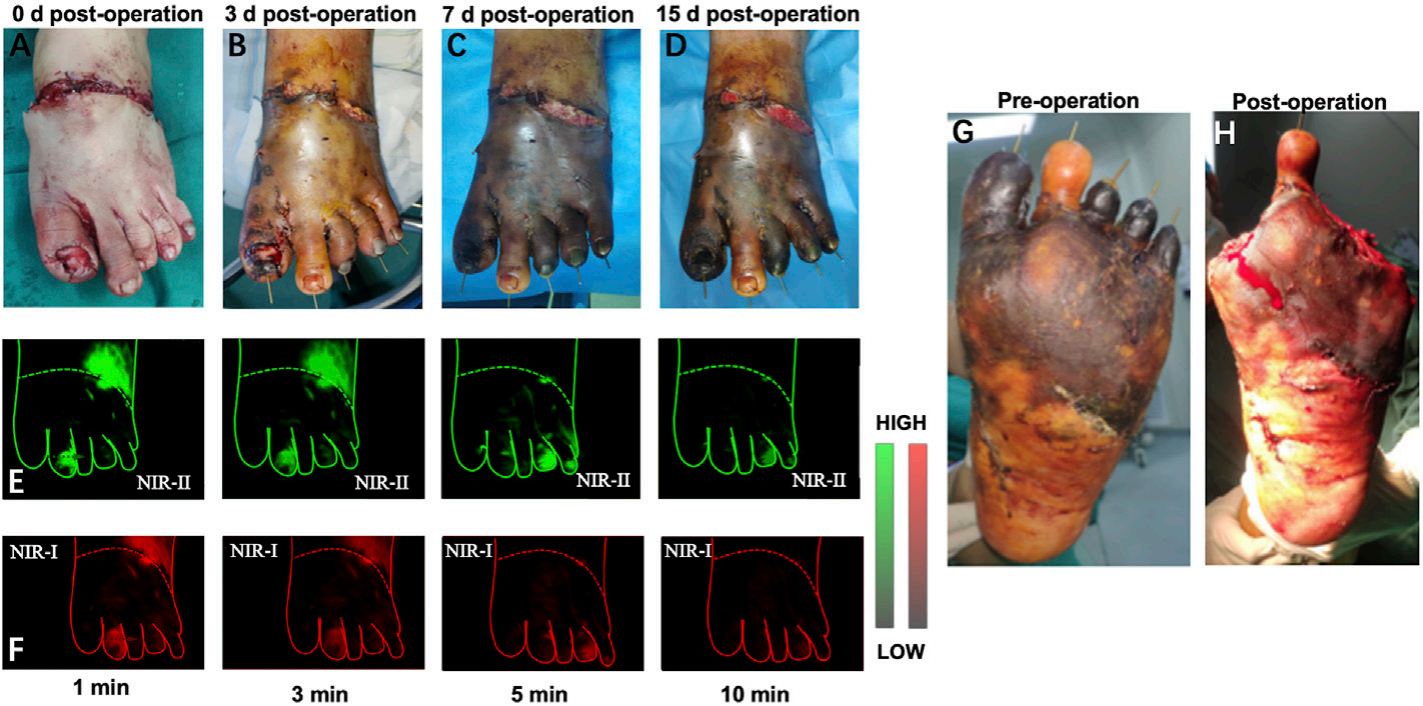
Example of a 29-year-old man received a two-stage foot skin avulsion injury debridement. **(A–D)** 0, 3, 7, 15 days after the first debridement. **(E,F)** Representative NIR-II and NIR-I window images for the assessment of arterial blood supply and venous reflux at different time points, respectively. **(G,H)** The second debridement was performed at day 15 after the first debridement.

In this section, we performed preoperative and postoperative fluorescence imaging for all five patients (representative angiography shown in Figure 4). In the presented case, the second toe of the degloving foot can be seen immediately after ICG injection and gradually metabolized in the third minute. The fourth and fifth toes did not start imaging until the fifth minute, which also took longer to metabolize. The first and third toes were not visualized the whole time. When observing immediately after debridement, the direct visual inspection did not see any difference in blood supply between these five toes, but with the passage of time, the obvious difference between the second toe and other toes could be seen on the seventh day after surgery. Finally, on the 15th day after the first debridement, the patient underwent a second stage debridement and removed the first, third, fourth and fifth toes. The decision-making of the second stage operation is well aided by the prediction of the NIR imaging.

### NIR imaging for the detection of skin perforators and evaluation of flap perfusion

**FIGURE 5:**
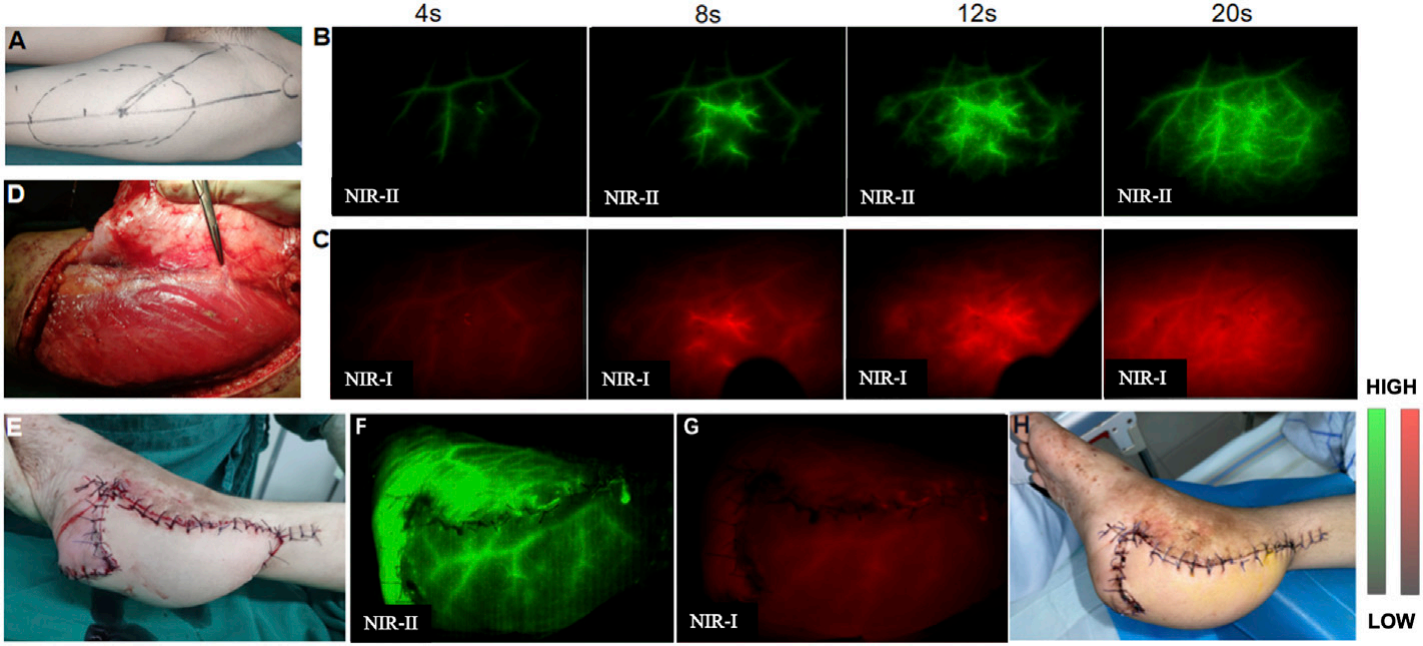
A 18-year-old man with the left heel scar contracture who underwent scar excision and reconstructive surgery by microsurgical free anterolateral thigh flap transfer. **(A)** One perforator was preliminarily located by Doppler sonography before performing NIR imaging. **(B,C)** Preoperative NIR-II imaging revealed four possible perforators, one of which overlapped with the Doppler location. **(D)** Perforators were found intraoperatively, and their locations agreed exactly with those observed by NIR imaging. **(E)** Flap transplantation was completed. **(F,G)** NIR-II imaging displayed satisfactory flap perfusion and clearer revascularization than NIR-I imaging after transplantation. **(H)** The flap showed complete viability and there were no complications 7 days postoperatively.

The reconstructed defects were in the regions of the upper and lower extremities and the trunk. The types of flaps were seen in Supplementary Table S1 and a representative process for microsurgical flap transplantation was shown in Figure 5. Preoperative NIR-II imaging detected more perforators than Doppler in all nine patients, and all these vessels were found at the deep fascial level during surgery. The skin flaps raised in all nine patients were island flaps for which the feeding vessels were limited to the perforator(s), and there was no arterial inflow from other sources. In contrast, although NIR-I imaging could display perforators at the same locations, the imaging is fuzzy with high auto-fluorescence of normal tissue and not as clear as NIR-II imaging, especially with the prolongation of injection time, the fluorescence of perforators was covered by normal tissue fluorescence and could not be distinguished by NIR-I imaging firstly. In assessing flap perfusion, NIR-II imaging was also clearer with high contrast than NIR-I imaging. The flap remained viable 7 days postoperatively in all nine patients. There were no adverse reactions to the infusion of ICG.

## Discussion

NIR-II imaging is turned to be a new rising technology in dealing with integrated diagnosis and image-guided therapy. Compared with NIR-I imaging, 1,000–1,700 nm NIR-II window guaranteed less autofluorescence, higher signal-to-background ratio (SBR), reduced photon scattering and lower levels of photon absorption, thus bringing NIR-II imaging a more promising technique in clinical environment (Wan et al., 2019). Previous researchers have reported several clinical trials NIR-II imaging in guiding the intraoperative hepatic carcinoma resection as well as suspected lymph nodes, for the enhanced permeability and retention (EPR) effect may play the most important role in this tumor tracking and therapy (He et al., 2019; Hu et al., 2020; Urade et al., 2020).

Compared with other imaging methods, NIR-II imaging has several advantages. Its real-time observation characteristics enable it to observe the arterial perfusion and venous reflux through the rate of change in fluorescence intensity. Not only it is non-invasive, it also has the advantages of zero contact with the patient and portability to change the position while the camera stays stable, meets the aseptic requirement in the operating room. Previous studies have reported the shortcoming of NIR-II/I imaging instrument and why it is not suitable for clinical use, such as the limitation of imaging speed, depth and width due to its original intention was made for small animals. Imaging system for large animals or human trials were rarely reported (Cai et al., 2020), most of the preclinical NIR imaging researches only included NIR-I imaging (Tanaka et al., 2014; Strübing et al., 2018). In this research, the portable NIR-II imaging system shows significant applicability to microsurgery in clinical situations.

The traditional evaluation for vascular anastomosis usually uses vascular patency test or ultrasound (Mücke et al., 2010). However, those two methods have the risk of damaging the vascular endothelium during contact, which may cause failure of anastomosis (Holm et al., 2009). These risks can be completely avoided by applying NIR imaging. In this study, 14 patients underwent microvascular anastomoses, each of them was assessed immediately after the vascular anastomosis. The process of bloodstream passing through the anastomotic sites were captured in real time in all cases, as an unquestionable mark, cross validated with the results from patency test. Follow-up observations confirmed the success of each operation, showed a latent and promising gold standard for future’s anastomosis evaluation.

Digit replantation is the complex technique of anastomosis on both artery and vein, arterial and venous obstruction can be detected during the operation *via* NIR-II imaging (Tang et al., 2020). But unlike simple vascular anastomosis, whether the replanted digit can survive or not also depends on sufficient arterial supply and compatible venous reflux. Traditionally, the color of digital skin and swelling condition are the reliable signs for clinical decision. As in this work, once the ICG was injected, the sooner the digit being fully fluoresced suggests that the arterial supply are more sufficient. Valuable time can be saved by refraining from anastomosis on another artery and still reach optimal surgical effect. The time interval between the digit being fully fluoresced and disappeared is related to the venous reflux efficiency. Prolonged fluorescence existing time indicates hypostasis and swelling, bloodletting therapy or anastomosis on another vein should be considered to relieve the load of veins. Intraoperative NIR-II imaging on the replanted digit can offer dynamic adjustment of surgical procedure and make a big difference for the prognosis.

Avulsed skin injuries frequently couple with crushing and rolling injuries, have greater area and multiple-plane injury. Therefore, it is very important for treatment option selection to evaluate the extent of the injury based on the assessment of micro vessels (Zhang et al., 2021a). The major benefit of NIR-II imaging in avulsed skin is providing reliable evidence for decision-making in debridement and reconstruction. Damaged micro-vessels make the injured area presented as delayed fluorescent or no fluorescent at all under NIR imaging, while the skin color shows no distinguishable difference. NIR-II imaging presents more distinct microvascular structures and higher resolution in perfused zones of avulsed skin than NIR-I imaging. Consequently, NIR-II imaging can predict skin necrosis, adjust debridement range, assuring aesthetic and functional results with higher precision.

Donor flap acquiring and replantation in recipient part are two major procedures in flap reconstruction, the design of flap requires precise identification of perforator artery (Onoda et al., 2013). Ultrasound and CTA are wildly applied preoperatively, but the low resolution of ultrasound and the inconvenience of that the CTA cannot be used synchronously in the operating room in real time limited their use (O’Malley et al., 2016). Better imaging quality is also achieved using NIR-II imaging, compared with NIR-I, benefited from the reduced tissue autofluorescence, reduced photon scattering and the low levels of photon absorption at longer wavelengths. With higher resolution and deeper detection range, NIR-II imaging could easily locate the ideal artery without the interfere of tissue autofluorescence. High resolution and high signal to noise ratio not only makes it more possible to locate the perforator vessels, and increased the accuracy of perforator border prediction.

We also found that the short half-life span (150–180 s) is ICG’s most adequate feature for microsurgery angiography (Bhavane et al., 2018). Within the safe dosage, ICG can be injected multiple times, which allows the surgeon to monitor the progress of the lesion’s circulation intraoperatively. However, it is expected that the performance of NIR-II imaging can be further improved with targeted probes that are purpose designed.

## Conclusion

The study indicates that NIR-II fluorescence imaging with ICG provides a non-invasive, high-resolution, deep penetration, large scale and real-time detection of the cutaneous microvasculature, which makes it a promising tool for the wide spectra of microsurgeries. Our study demonstrates that NIR-II imaging has advantages over traditional fluorescence imaging in clinical scenarios, and thus highlights the promising clinical potential of intraoperative NIR-ΙΙ fluorescence imaging and NIR-II image-guided surgery.

## Data Availability

The raw data supporting the conclusion of this article will be made available by the authors, without undue reservation.
