# Supplementary material for: First clinical applications for the NIR-II imaging with ICG in microsurgery
Source: Front Bioeng Biotechnol. 2022 Oct 18;10:1042546. doi: 10.3389/fbioe.2022.1042546 (PMC9623121; doi:10.3389/fbioe.2022.1042546)
Supplement: Supplementary file 1 [file Table1.DOCX]

Table S1 Summary of characteristics

| Age (y) | 34±12 (19–52) |
| --- | --- |
| Sex, n | Male: 32  Female: 7 |
| Injuried vessel, n | Radial artery：3  Popliteal artery：4  Ulnar artery：1  Superficial palmar arch：1  Femoral artery：1  Dorsalis pedis artery：1  Posterior tibial artery：2  Posterior tibial vein :1 |
| Injured digit, n | Thumb: 5  Index: 4  Long: 2 |
| Injured skin avulsion, n | Foot: 2  Arm: 2  Leg: 1 |
| Type of Flap, n | Anterolateral thigh: 3  Anterior tibial artery perforator: 2  Inferior epigastric artery perforator: 1  Internal thoracic artery perforator: 1  Posterior tibial artery perforator: 2 |

Note: Data are presented as mean±standard deviation (range) unless otherwise indicated.
